# Supplementary material for: Are carbon emissions trading and green financial instruments synergistic? -Comprehensive quantitative research based on content analysis
Source: PLoS One. 2024 Mar 7;19(3):e0298601. doi: 10.1371/journal.pone.0298601 (PMC10919601; doi:10.1371/journal.pone.0298601)
Supplement: S1 Data — (ZIP) [file pone.0298601.s001.zip › Data/地级市Z_F工作报告.zip!/╔╜╢1⁄2/╝├─■╩╨/└┤╫╘╣1⁄2╓┌║┼í╛┬φ┐╦╩2╛▌═°í┐_2006.docx]

各位代表：

　　现在，我代表济宁市人民政府，向大会作工作报告，请予审议，并请市政协委员提出意见。

　　一、“十五”计划主要预期目标提前实现，济宁站在一个新的发展起点上

　　新世纪的头五年，我市改革开放和现代化建设取得显著成就，“十五”计划主要预期目标提前一年实现，市十四届人大三次会议确定的年度各项任务全面完成。

　　（一）经济持续快速增长，综合实力跃上新台阶。2005年全市地区生产总值达到1266.2亿元，比上年增长17.3%，比“九五”末翻了一番。人均地区生产总值1956美元，比“九五”末增长1.2倍。地方财政收入66.5亿元、增长23%，比“九五”末增长1.39倍。固定资产投资667.1亿元、增长31.3%，比“九五”末增长2.8倍。金融机构年末存款余额939.57亿元、贷款余额623.14亿元，比“九五”末增加528.45亿元和306.65亿元。

　　（二）经济结构日趋优化，产业层次获得新提升。三次产业比例由“九五”末的20.8:43.6:35.6调整到2005年的13.7:55:31.3。农业五大产业体系建设取得阶段性成果，林牧渔业产值占比提高到46%。全市规模以上工业企业达到1559家、比“九五”末增加1038家，利税过千万元工业企业149家、增加89家。重汽商用车、山推国际事业园、小松叉车、德源纱厂、嘉达纺织、凯赛生物、明治鲁抗、胜利制药、伊顿液压、太阳纸业技改扩能、易路轮胎、科澳铝业、泰山玻纤、金皇活塞、金威电化、东岳专用车等一批“高大外”项目竣工投产，建成工程机械、生物技术、纺织新材料三大国家级产业基地，创出6个中国驰名商标、43个省著名商标和53个全省全国名牌产品，高新技术产业产值占工业总产值的比重达到22.4%，比“九五”末提高14个百分点。大型商贸流通企业通过改造重组焕发新的活力，连锁、超市、物流配送等新型业态加速发展。我市跨入“中国优秀旅游城市”行列，2005年接待中外游客1234万人次，旅游社会总收入81.5亿元，比上年增长30%。民营经济增势强劲，完成增加值533.9亿元，比上年增长34.4%，上缴税金54.7亿元、增长37.1%。统计执法和工商、物价监管力度持续加大。

　　（三）改革迈出较大步伐，对外开放实现新跨越。骨干企业规范化股份制改造进度加快，首批21家国有及控股企业纳入市国资委监管体系。政府机构和行政审批制度改革积极推进。事业单位改革开始启动。农村税费改革成果进一步巩固，综合配套改革进入试点阶段。投资、财税、金融和粮食流通体制等改革稳步实施。500千伏输变电工程投入运行，长期制约我市发电企业的“窝电”问题得到解决。15个开发区获得上级核准。一批水利、交通、航运等基础设施及采煤塌陷地治理项目进入国家、省专项计划。先后两次争取到提高煤炭资源税单位税额的调整政策，增加了可观的地方财力。6个经济薄弱县通过优化结构、做实收入获得专项扶持资金1.6亿元，使编制内财政供养人员按国家标准发工资的目标提前实现。成功举办金融生态环境建设暨银企合作促进会，与24家市外金融机构签署262.8亿元合作协议，去年到位资金98亿元。国际孔子文化节招商平台作用日益凸显，组团参加境内外招商活动成果丰硕。2005年引进市外国内资金201亿元，外商直接投资2.48亿美元、增长68.4%，完成外贸进出口总额18.68亿美元、增长29.3%，其中出口11.6亿美元、增长41.1%。全市经济外向度由“九五”末的5.5%提高到12%。

　　（四）城乡建设力度加大，人居环境有了新改善。编制完成新一轮城市总体规划和一批专业规划。城市化水平达到40.5%，比“九五”末提高6.2个百分点。五年新建改造公路7600公里，高速公路里程增加到183公里，327国道城区段改线工程加快建设，崇文大道、日东高速济宁连接线及105国道改造等城际间公路干线建成通车。“绿亮清”工程五年累计投入46.3亿元，建成一批广场、绿地及标志性建筑。2005年完成3条市区主干道和25条小街巷新建改造任务，河湖水系综合整治工程启动建设。新增集中供热面积60万平方米，天然气引进正在加紧施工。城区绿化率达到35.4%，我市荣获省级园林城市称号。8处城市污水处理厂建成运营。济宁城市生活垃圾处理厂开工建设。城乡环境综合整治初见成效。城市经营性用地先详规再招拍挂制度全面落实。生态市建设规划深入实施，工业点源污染和农业面源污染得到有效治理，封停城区供水管网内自备井84眼，南四湖人工湿地建设扩大到3000亩。30家燃煤电厂完成脱硫改造任务，化工发酵企业异味污染得到有效遏制，城区空气质量优良率达到90%以上。

　　（五）社会事业全面进步，人民生活水平得到新提高。五年累计完成重要科技成果730项，其中118项获省以上科技进步奖，申报专利2715件、授权1511件。连年成功举办中国专利高新技术产品博览会和“院士济宁行”活动。建成44处产学研基地、3处博士后工作站，省级工程技术中心发展到26家。“普九”成果巩固提高，省级规范化学校增加到62处。职业技术教育在资源整合中创新发展。济宁师专迁入新校区，申报济宁学院通过省级评估验收。“中华文化标志城”前期工作有效推进。一批文艺精品在全国、全省获奖。公共卫生“两大体系”建设基本完成，市疾控中心挂牌成立，新型农村合作医疗试点扩大到6个县市区，惠及197.3万农民群众。高致病性禽流感防控工作扎实开展。我市体育健儿在十运会上获得好成绩。连年完成省下达人口与计划生育工作责任目标。新闻出版、广播电视、民族宗教、外事侨务、人防地震、水文气象、档案史志、老龄和残疾人等事业获得新的发展。2005年实施村村通自来水工程173项，解决50万群众安全饮水问题。建成农村公路2100公里。改造农村中小学校舍危房5万平方米，救助贫困生1.53万人。建设经济适用房10.29万平方米，市区319户困难居民迁入廉租房。新增城镇就业11万人，登记失业率3.2%。全市养老、失业、医疗、工伤保险参保人数新增10.8万人、达到216.8万人。市县财政投入低保资金5400多万元，4万多户城乡低保对象生活得到保障。全市城镇居民人均可支配收入10739元，农民人均纯收入4128元，分别比上年增长13%、13.2%。市政府承诺为民所办10件实事如期完成。

　　（六）民主法制建设加强，文明创建取得新成果。及时向人大及其常委会报告工作、向人民政协通报情况，认真办复人大代表建议、政协委员提案。坚持依法行政。保持共产党员先进性教育活动成效明显。完成“四五”普法和第八届村民委员会换届直选。政务公开、村务公开、厂务公开制度逐步完善。开通“12345”“市长公开电话”。建立了政府新闻发言人制度。强化审计监督和行政效能监察。严格执行反腐倡廉各项规定。“文明济宁”、“平安济宁”、“和谐济宁”建设扎实推进，15个单位获得全国文明单位和文明创建先进单位称号，我市荣获“全国双拥模范城”四连冠、全省创建文明城市先进单位。社会治安防控网络日趋完善，矛盾纠纷调处机制进一步健全，安全生产形势总体平稳。

　　各位代表，“十五”的发展成就，归功于省委、省政府和市委的正确领导，得益于历届领导班子打下的良好基础，离不开市人大、市政协的监督支持，凝结着全市广大干部群众的心血和汗水。在此，我代表市人民政府，向全市广大工人、农民、知识分子和干部，向各民主党派、工商联、人民团体和各界人士，向中国人民解放军驻济部队指战员、武警官兵和公安干警，向中央、省驻济单位，向来济宁创业发展的各位投资者和建设者，向关心支持济宁现代化建设的港澳台同胞、海外侨胞和国际友人，表示衷心感谢，致以崇高敬意！

　　我们也清醒地看到，经济社会生活中的困难和问题还不少。经济结构性矛盾依然突出，煤炭产业占比过大的状况没有根本改变，服务业、高新技术产业和对外贸易比重偏低。农业综合生产能力和效益不够高，农民持续增收难度加大。自主创新能力不强，经济增长方式比较粗放。市场取向改革深度不够，困难企业改制脱困进展迟缓。县域经济发展不平衡，特别是工业基础较为薄弱。农村社会事业发展滞后，就业、社保和人口、资源、环境面临较大压力，涉及群众切身利益的一些问题还没有得到很好解决。各级政府职能转变不快，一些工作落实不够，办事效率不高，不作为、滥作为现象时有发生，影响发展环境的问题比较突出。我们要进一步增强使命感和紧迫感，以昂扬向上的斗志、奋发有为的精神和求真务实的作风，努力把政府各项工作做得更好，不辜负人民的厚望与重托。

　　二、围绕“十一五”发展目标，扎实做好开局年的各项工作

　　从今年开始，我们步入“十一五”发展新阶段。按照中央《建议》和省、市委部署要求，市政府编制完成了《济宁市国民经济和社会发展第十一个五年规划纲要（草案）》，在系统总结“十五”计划实施情况，透彻分析未来五年发展走势与宏观环境的基础上，提出了“十一五”期间全市经济社会发展的目标任务。《纲要（草案）》及其《说明》已印发大会，请一并审议。

　　《纲要（草案）》确定的“十一五”我市经济社会发展的总体要求是：以邓小平理论和“三个代表”重要思想为指导，深入贯彻党的十六大和十六届三中、四中、五中全会精神，以科学发展观统领全局，按照省委“一二三四五六”的总体工作思路，围绕建设经济强市、文化名市、组群结构大城市目标，坚持“全党抓经济，重点抓调整”，坚持举全市之力扩大开放，坚持工业化与城市化双轮驱动，增强自主创新能力和体制机制活力，大力推进经济结构优化升级、增长方式根本转变和城乡统筹协调发展，加快建设社会主义新农村，努力构建和谐济宁、生态济宁，不断提高人民群众生活水平，开创科学发展、加快发展新局面，为提前建成全面小康社会、提前基本实现现代化奠定坚实基础。综合考虑各种因素，《纲要（草案）》从经济增长、改革开放、协调发展、和谐社会、资源环境、人民生活等方面，提出了“十一五”发展的主要预期目标，其中全市地区生产总值年均增长13%以上，2010年达到2330亿元，人均占有3800美元；地方财政收入年均增长15%，比“十五”末翻一番，达到135亿元；固定资产投资年均增长20%左右，五年累计完成5950亿元；单位生产总值能耗比“十五”末降低23%左右。《纲要（草案）》从七个方面安排了“十一五”发展的战略任务，对建设社会主义新农村、加快结构优化升级和增长方式转变、深化改革扩大开放、推进区域经济协调发展、建设资源节约型环境友好型社会、大力实施科教兴市和人才强市战略、以人为本构建和谐社会等重点工作，提出了明确的阶段性目标和政策措施。《纲要（草案）》经本次大会审查批准后，我们将分解目标，落实责任，细化措施，认真组织实施，确保如期完成各项既定任务。

　　今年是实施“十一五”规划的第一年。我们要按照省、市委的部署要求，全面落实科学发展观，坚持推进经济结构调整、改革开放、自主创新和增长方式转变，努力保持经济平稳快速发展；统筹抓好城乡建设，更加注重社会事业全面进步；认真解决关系群众切身利益的实际问题，加快构建和谐社会，确保实现“十一五”发展良好开局。今年全市经济社会发展主要预期目标是：地区生产总值增长14%，万元生产总值能耗降低5%，地方财政收入增长17%，固定资产投资增长25%，外贸进出口增长25%、其中出口增长30%，外商直接投资增长35%，城镇居民人均可支配收入和农民人均纯收入均增长8%，城镇登记失业率控制在4%以内，人口自然增长率控制在6.5‰以内。 今年要着力抓好十个方面的工作。

　　（一）扎实推进社会主义新农村建设。按照中央确定的20字目标要求，贯彻工业反哺农业、城市支持农村的方针，加大对“三农”支持力度。从实际出发，因地制宜，分类指导，搞好规划。尊重广大农民意愿，发挥农民主体作用。自力更生，艰苦奋斗，真抓实干，务求实效。

　　加快生产发展。今年市级财政安排“三农”支出2.4亿元，比上年增长45.17%。继续调整优化农业结构，围绕拉长“五大产业链”，规划建设一批规模化、标准化种养基地，大力兴办农村二三产业特别是农产品加工业，规范发展各类合作经济组织和农村经济协会，推进农村劳动力向非农产业和城镇有序转移。增强农业科技创新和转化能力，加强农业技术推广和服务。加快兽医管理体制改革和动物疫病防控体系建设，扎实做好高致病性禽流感防控工作。从兴办配套协作项目、盘活各类集体资产等入手，探索新形势下村级集体经济发展的有效途径。立足改善农业生产条件，坚持不懈地开展农田水利基本建设，重点推进灌溉、道路、电力等基础设施向种养基地延伸配套。

　　促进生活宽裕。千方百计拓展增收渠道，引导农民发展优质高产高效粮食作物，扩大高价值经济作物和畜禽、淡水产品饲养规模。强化农村劳动力技能培训，配套建设县乡劳动力市场，力争有劳动能力的农户至少一人外出务工。落实粮食直补、粮种补贴、农机具补贴等项支农惠农政策，今年起全市范围内全部免征农业税。

　　倡树乡风文明。以提高农民素质为着力点，大力实施系列教育培训工程。增加公共财政对农村各项社会事业的投入，今年市级财政安排农村义务教育经费1015万元，比上年增长34.43%。从今年起用两年时间，全部免除农村义务教育阶段学生学杂费，对贫困家庭学生免费提供教科书并补助寄宿生生活费。及时改造校舍危房，年内解决部分学校学生带桌凳上学问题。各县市区全部推行新型农村合作医疗试点，参合农民达到80%以上，按规定提高补助标准，其中市级财政安排补助资金3019万元。积极推进农村“文化大院 ”建设。抓好农村社区综合服务中心创建工作。坚持实行农村计划生育家庭奖励扶助制度。

　　务求村容整洁。深入开展镇村环境综合整治，集中抓好卫生保洁、绿化美化、监督管理，乡镇驻地要率先改善面貌，发挥示范带动作用。巩固发展农村“文明一条街”建设成果，实施“清洁家园工程”，年内10%的行政村完成“一池三改”，积极推广太阳能和秸秆综合利用。结合压煤村庄搬迁和城市近郊村庄整理，规划建设小康村示范点。加快村村通自来水工程建设，今年全市农村自来水普及率达到75%以上。以加快向自然村延伸贯通为重点，新建乡村公路500公里。

　　推进管理民主。深入开展保持共产党员先进性教育活动。建立健全村“两委”班子议事规则和工作运行机制，修订完善村规民约，严格村民自治制度，规范村务公开内容与形式，健全村级服务组织，保证社会治安、计划生育、环境整治、公用设施建设等职责落实到位。

　　（二）大幅提高经济增长外向度。在更高层次上扩大对外开放，创新招商方式，增强招商实效，优化出口商品结构，扩大对外贸易规模。

　　全力突破外资大项目。从企业招商上突破，指导利税超千万元企业搞好项目推介和对接洽谈，拿出优质资产与国内外大企业特别是世界500强合资合作，靠引进外资发展壮大。引导外资企业着眼拉长产业链条，在增资扩股的同时，积极引进海外企业上下游配套项目，争取承接主导产品生产线的整体转移。从园区招商上突破，督促省级开发区配套完善基础设施，创新管理运行机制，提升承载功能和服务水平，面向重点国家、地区组织开展富有成效的招商活动，力求尽快形成外资企业集群。济宁高新区集中引进基地型、龙头型、高端型外资大项目，争取尽早跨入国家级高新区行列。从节会招商上突破，放大孔子文化节品牌效应，着力创新办节方式，突出招商主题，把握会前广泛洽谈、会上集中推进、会后跟踪落实三个环节，进一步提高招商实效。从基础设施和服务业招商上突破，加快运作落实城市污水处理及供水供气合资合作项目，力求现代物流、旅游和文化产业招商开发尽快破题。重视扩大对内开放，面向市外发展经济技术交流合作。

　　积极转变外贸增长方式。努力提高机电产品、高新技术产品出口比重，培植壮大出口骨干企业群体，促进加工贸易转型升级。集中引进重要原材料、关键技术和设备。支持有条件的企业实施“走出去”战略，争取在海外战略资源开发、生产基地建设、承包工程和外派劳务方面有大的进展。

　　切实优化开放环境。着力营造高效便捷的政务环境，严格兑现“只要项目定了干，一切手续我来办”等服务承诺，建立完善“一个项目一名领导、一套班子、一条龙服务”的工作机制，保证项目审批建设“一路绿灯”。着力营造公平安全的法制环境，完善促进外资项目建设有关政策，细化落实项目施工建设包保措施。及时处理投诉案件，严厉查处侵害投资者合法权益的不法行为。着力营造舒适宜人的生活环境，有条件的地方要规划建设外商集中居住区，配套完善服务设施，解决好外商休闲、就医、家政服务、子女入学等实际问题。着力营造亲商安商的社会环境，广大干部群众必须强化“人人都是投资环境、事事关系济宁形象”的责任意识，齐心协力为扩大开放办实事、办好事，真正让投资者放心、安心地在济宁创业发展。

　　（三）集中力量打造制造业强市。立足做大做强工业经济，下更大气力调整优化产业结构，加快建设五大产业基地，着力培育制造业发展的规模优势、技术优势和品牌优势，尽快改变资源型产业占比过大的状况。一是建设煤化工产业基地。发挥兖矿集团的龙头带动作用，有机整合市内煤炭企业要素资源，围绕煤焦化、煤气化、煤液化三大链条，主攻“醇、苯、酸、烯、油、肥”系列产品开发。煤矿企业都要落实“一矿一区”精深加工项目，构建煤矿园区良性互动的发展格局，逐步形成横向成峰、纵向成链的煤化工产业集群。二是建设机械制造产业基地。依托山推股份、中国重汽商用车、山东东岳等骨干企业，做大重型卡车、推土机、挖掘机、叉车、专用改装车等整机产品生产规模，兴办专业协作项目，提高产业配套能力和集中度。三是建设生物技术产业基地。以凯赛、菱花、雪花等重点企业为主导，转化最新科技成果，加快建设合资项目，主攻生物制品、生物材料、生物能源等领域，集中开发生产赖氨酸、氨基酸、长碳链二元酸、燃料乙醇等系列产品。四是建设医药食品产业基地。加快抗生素系列产品换代升级，发展高科技含量、高附加值的生物医药产品；立足农副产品精深加工，积极开发新兴系列食品，培育特色优质品牌，不断提升食品产业档次水平和规模效益。五是建设纺织服装产业基地。以如意、樱花、德源、嘉达、欧化日棉等企业为主体，发展高支精梳纱，研制生产多功能面料，提高印染后整理水平，开发中高档西服、时装、休闲装及床上用品，精心培育国内一流服装品牌。对造纸建材等产业实施系统改造和战略重组，重点发展高档纸制品和新型建材。从培植高新技术企业入手，促使机电一体化、电子信息、新材料等新兴产业加速成长。今年全市高新技术产业占规模以上工业产值比重提高2个百分点以上。围绕建设五大产业基地，继续实施大项目带动，集中力量加快建设144个亿元以上产业项目；扎实推进创百亿工程，年内销售收入过50亿元企业达到4家，新增利税超千万元企业30家；强化质量兴市，支持骨干企业争创省以上名牌产品。

　　（四）激发服务业发展活力。认真落实发展服务业各项扶持政策，进一步拓展领域、创新业态、优化结构、扩张规模，提高对全市经济和就业的贡献率。

　　改造提升流通业。整合传统商业资源，大力发展连锁、超市、配送和现代批发市场等新型业态。引进国内外大型商贸企业参与市内流通企业改制重组。加快供销企业转型，构建农村新型流通服务网络。着力开发生产性服务业项目，科学规划建设现代物流中心，支持大型骨干企业兴办第三方物流。大力发展金融保险业，组建济宁市商业银行，引进更多市外金融机构来济宁合作发展。支持各商业银行开发金融服务新品种，拓展银企合作新路子，持续增加信贷投放规模。积极创办中介咨询、社区服务、物业管理、教育培训、医疗保健等新兴服务业项目。

　　加快发展旅游业。认真实施《济宁市旅游发展规划》，突出抓好旅游资源深度开发，打造一批人文景观、山水风光、民俗风情、休闲度假等重点旅游项目。加快旅游宾馆等服务设施建设。强化旅游从业人员管理培训，提高接待服务水平。开发健康、文明、参与性强的娱乐项目，延长游客停留时间，增加旅游消费。抓好旅游环境综合整治，规范旅游市场秩序。抓好旅游客源市场开发，利用海内外主流媒体打造“孔孟之乡”旅游品牌，实现我市精品旅游线路与周边城市的有机对接、客源共享，年内接待中外游客1400万人次，旅游社会总收入达到100亿元。

　　积极培育文化产业。规划建设市体育中心、图书馆、济宁大剧院等场馆，在城市社区和乡镇配套建设文化体育设施。深化文化体制改革，落实相关扶持政策，有序推进经营性文化事业单位转企改制，培育组建广电、报业、演艺和印刷出版等领域的重点文化企业集团。借助山东省首届文化产业博览会平台，包装推介一批文化产业项目对外招商。积极探索文企联姻新路子。

　　（五）发展壮大县域经济。鼓励经济强县放大优势、率先发展，把功夫下在高起点推进结构调整、增强自主创新能力、扩大对外开放、培植优势产业和骨干企业集群上，不断提升经济整体素质和竞争实力，在科学发展、加快发展、和谐发展上走在全市全省前列。促进中间县自加压力、加速崛起，重点是增加有效投入，兴办新型工业项目，壮大特色主导产业，在夯实基础、膨胀规模、增强后劲上见到大的成效，争取综合实力排名在全省位次前移。激励经济薄弱县知难而进、跨越发展，着力在用好扶持政策、增强内生动力、主攻工业经济、大办创税项目上加大力度、有所作为，力争经济总量、投资规模、可用财力三项指标增幅高于全市全省平均水平，尽快走出困境。以实施“百家中小企业成长计划”为抓手，优化系列服务，支持全民创业，引导民营企业创新发展模式，提升档次水平，培植立县立乡大企业。年内每个县市区新增规模以上工业企业40家，经济强县力争多上大项目、多办大企业，经济薄弱县要充分挖掘自身潜力，创办更多规模以上企业。

　　（六）切实增强自主创新能力。围绕建设创新型企业、创新型社会和创新型城市，坚持把引进消化吸收再创新作为自主创新的主渠道，重点抓创新体系建设，在骨干企业建立完善研发机构，年内新增省级以上工程技术中心5家，争取建成1—2家国家级工程技术中心。依托济宁高新区办好创业中心，发展专业型孵化器。抓成果引进转化，提升中国专利高新技术产品博览会档次和外向度，深入开展“院士济宁行”活动，争取新建中试基地和“引进国外智力示范园”。支持各类企业采取购买专利、难题招标等形式，转化吸收最新科技成果，提高装备工艺水平。年内争取开发高新技术产品260项，新增高新技术企业80家。抓联合集中攻关，围绕主导产业发展特别是五大产业基地建设，整合市内企业及驻济高校、科研院所研发力量，开展共性、关键、核心技术联合攻关，争取在煤炭精深加工、生物医药等领域率先开发一批拥有自主品牌的新产品，在节能降耗、废物利用、污染防治等方面破解技术瓶颈。强化知识产权保护管理。探索发展风险投资。

　　围绕增强自主创新能力，加快建设人才高地。发挥驻济高校和各类职业技术院校优势，为重点行业、骨干企业定向培养技能型人才。舍得花钱引进对外经贸、经营管理、自主研发、城市规划建设等方面的创新型人才。规范完善技术、管理等要素参与收益分配的政策，鼓励支持学科带头人和专利持有人来我市创业发展。对创新发展贡献突出的企业家、科研人员给予重奖。

　　（七）加快体制机制创新。扎实推进各项改革，争取在一些重要领域和关键环节取得突破性进展。坚持分类指导抓好事业单位改革，年内基本完成市属社会公益类事业单位改革任务。强化人事编制管理，严格执行有编制、有计划、有预算和逢进必考、优中选优的制度规定，切实把住进人关口。从试点入手抓好农村综合配套改革，力求在整合精简乡镇机构、妥善分流超编人员、改进事业站所服务功能、减轻财政支出压力等方面率先见效。因企制宜抓好企业改革，加快骨干企业规范化股份制改造，完善法人治理结构。坚持一厂一法、合力攻坚，促使困难企业尽早实现改制脱困。按照管资产与管人管事相统一的原则抓好国有资产管理体制改革，落实国有资本出资人制度和经营预算制度，完善监管考核体系，调整优化国有经济布局和企业组织结构，促使监管企业做大做强。以建立公共财政框架为目标抓好财税体制改革，积极推行部门预算、国库集中支付制度，扩大政府采购范围，加快实行“乡财乡用县管”。健全社会综合治税机制，保证各类税收足额入库。稳步推进国有商业银行改革，继续深化农村信用社改革。

　　（八）加大城市规划建设管理经营力度。坚持城乡统筹、以人为本的原则，按照高起点规划、高质量建设、高效能管理、高水平经营的要求，打造更多城乡建设亮点。

　　按照超前、适时要求抓规划。依据新一轮城市总体规划，加快编制专项规划，及时满足建设需要，尽早实现重点区域控制性详细规划全覆盖。加紧编制镇村规划，强化对新农村规划建设指导，向农民群众无偿提供新型住宅设计样图。搞好规划、建设、综合执法等部门协调配合，依法查处乱搭乱建等违法违规行为。

　　按照优质、高速要求抓建设。重点抓好城市基础设施建设。济菏高速公路济宁段、327国道改线及一批国省道改造工程年内竣工通车。优化京沪高速铁路建设环境。加紧建设军民合用济宁机场。做好济徐高速济宁段、京杭运河济宁至东平湖三级航道复航前期工作。立足挖掘文化内涵、体现区域特色、提升品位形象，扎实推进济宁城区“绿亮清”工程，规划建设一批标志性建筑。优化河湖水系综合整治施工方案，加快建设进度，打造清新灵动、风格别致的沿河景观带，展示“运河之都”风采。加快北湖旅游度假区开发建设，完善基础设施，增强服务功能，为市民营造休闲娱乐好去处。高起点招商开发城北片区，务求形成城市建设的新亮点。着眼打通交通瓶颈、提高通行能力，完成一批城区道路改造建设任务。明确市中区、任城区职责权限，加快改造城中村。完善城市建设重点项目推进机制，实行规划审批与工程建设同步监管，保证审批项目及时开工、在建项目尽早竣工。依法规范工程招投标，运用市场机制提高拆迁效率、降低拆迁成本。引导建设单位集中优势力量，改进施工方式，提高施工效率。

　　按照严格、精准要求抓管理。着眼于建立横向到边、纵向到底、全面覆盖的城市管理网络，合理划分市区管理事权，促使管理重心下移，在街道、社区推行网格化管理和居民自我管理，形成“两级政府、三级管理、四级网络”的管理格局。加强综合执法能力建设，及时查处各种影响城市容貌与秩序的违规行为。改进环卫作业方式，完善门前“三包”制度，突出抓好城乡结合部、城市出入口、车站、广场及背街小巷等重点区域、部位的环境综合整治与管理，在城区创建一批“管理示范街”。

　　按照开放、高效要求抓经营。完善土地收购储备机制，实行供应规模总量控制，努力实现土地资产收益最大化。坚定不移地放开城市开发建设市场，严格竞标企业资格审查和刚性条件，大力引进资质高、信誉好、实力强的投资者参与城市建设。按照产业化经营、企业化管理的要求，加快推进市政公用事业改制转机，探索城市道路、桥梁、广场、绿地等基础设施建设管理市场化的有效办法和途径，对一些公用设施的经营权、冠名权实施公开招标拍卖，多渠道增加城市经营收益。

　　（九）着力建设资源节约型、环境友好型社会。认真实施《济宁生态市建设规划》，遵循“减量化、再利用、资源化”的原则，争取在减少资源原料消耗、减少废弃物排放、减少环境污染及生态保护与重建等方面大见成效。

　　推行节约型增长方式。突出抓好节能、节水、节地、节材和节约矿产资源措施落实。集中在煤炭、电力、化工、建材、造纸、医药6个重点耗能行业和100家重点耗能企业推广节能新技术新工艺新装备，加快淘汰消耗高、污染重的落后生产能力，争取万元工业产值综合能耗年内降低8个百分点。修编完善新一轮土地利用总体规划，制定落实节约集约用地强制性标准，提高各类园区项目容积率和单位土地投入产出率。抬高矿产资源开发市场准入门槛，监督煤炭企业严格按核定能力组织生产，坚决杜绝掠夺式开采行为，确保煤矿回采率年内提高2个百分点。

　　加快发展循环经济。组织企业大力推行清洁生产，广泛开展ISO14000环境管理体系认证，培植10家循环经济先导示范企业。选择部分园区和产业关联度高的重点行业，搞好行业之间、企业之间的生产协调与组合，努力形成上下游配套成龙、原料产品循环利用、废弃污染物零排放的绿色产业集群。

　　加大污染防治力度。监督污水排放企业按照南水北调调水水质要求深化治理，同步抓好回收利用，确保2007年按调水标准排放，力争特定区域和部分行业实现污水零排放。加强水资源管理保护，继续封停城市供水管网内自备井。完成3处在建城市污水处理厂建设任务，保证已建成污水处理厂正常运营。加快中水回用等配套管网建设，开工建设截污导流工程。抓好农业面源污染防治。建成济宁城市生活垃圾处理厂。完成燃煤电厂年度脱硫改造任务，同步落实新建电厂脱硫装备。有效治理化工发酵企业异味污染，全年空气环境质量优良率保持在90%以上。构建全覆盖、全天候环境质量在线监测网络，严厉查处违法排污行为。认真执行环境影响评价和“三同时”制度，防止新污染源产生。

　　强化生态保护与重建。集中人力物力财力，卓有成效地开展采煤塌陷地治理会战，年内完成3万亩治理任务。落实各类生态功能保护区建设管理措施，扩大南四湖人工湿地面积。深化小流域综合治理，广泛开展植树造林活动，加快荒山绿化和绿色通道建设，年内全市林木覆盖率提高到24%。

　　（十）积极构建和谐社会。牢记执政为民宗旨，切实解决广大群众最关心、最直接、最现实的利益问题，做好就业、社保、安全生产等方面的工作。

　　努力促进充分就业。把扩大就业作为发展的优先目标，千方百计开辟就业岗位。认真落实就业再就业扶持政策，确保城镇“零就业家庭”至少一人实现稳定就业。做好大中专毕业生就业和退役军人安置工作。建立完善城乡一体化的人力资源市场，向农村劳动力提供平等竞争、自主择业的机会。今年全市新增城镇就业8万人，新增农村劳动力转移就业20万人。

　　完善社会保障体系。严格落实劳动合同制度，依法抓好养老、失业、医疗、工伤等社会保险扩面征缴，力争参保人数和基金征缴同步增长。增加各级财政低保投入，依托社区建立完善城镇低保动态管理网络，扩大农村低保覆盖面。完善失地农民基本生活保障制度。健全农村五保供养“四级联保”机制，年内集中供养率提高到70%。市区开工经济适用房10万平方米，及时为城镇“双困户”解决好廉租住房。继续对城乡贫困学生实施救助。加强防灾救灾工作。落实军烈属优抚政策。整合捐赠救助资源，积极发展社会福利和慈善事业，使失去父母的儿童、没有生活来源的老人和残疾人，得到更多的关爱与帮助。围绕群众迫切要求解决的困难问题，继续为民办好10件实事。

　　加快发展社会事业。增加各级财政的教育投入，深入推进义务教育“双高普九”，强化农村和城镇薄弱学校建设，年内创建省市级规范化学校30所。整合职业技术教育资源，发展与市内外大企业的战略协作。济宁学院申报通过国家验收。着力优化教学科研环境，支持驻济高校立足济宁加快建设发展。完成文化名市年度建设任务。有效推进中华文化标志城前期工作。提高广播电视、新闻出版、社会科学事业发展水平。完善文博、史志、档案社会服务功能。围绕树立以“八荣八耻”为核心的社会主义荣辱观，深入开展思想道德建设和群众性文明创建活动。扎实推进双拥共建，加强国防教育和民兵预备役建设，争创省级文明城市和全国双拥模范城“五连冠”。加快36个中心镇卫生院改造建设，发展城市社区卫生服务，推进中医现代化。强化药品、食品安全监管，整顿规范药品流通秩序，引导各类医疗机构学习济医附院的先进经验，落实便民利民服务措施，努力解决群众看病难、看病贵问题。开展全民健身活动，提高竞技体育水平，力争21届省运会再创佳绩。积极应对第四次人口出生高峰，有效治理出生人口性别比偏高问题，提高人口出生质量。保障妇女儿童权益。重视做好民族宗教、外事侨务、对台和老龄工作。提高气象、人防、地震监测预报水平。

　　深入创建“平安济宁”。完善社会治安群防群控体系，强化基层基础工作，集中整治治安落后单位，持续开展严打整治、打霸除恶斗争，坚决扫除“黄赌毒”等社会丑恶现象。启动实施“五五”普法，积极开展法律援助。健全矛盾纠纷调处机制，层层落实信访责任制。加强社会应急管理，提高处置突发事件的能力。严格落实安全生产责任制，强化企业安全生产主体责任，坚持不懈地开展隐患排查和安全生产专项整治，完善安全生产技术规范和质量工作标准，充分发挥职工群众对安全生产的参与和监督作用，标本兼治，重在治本，确保安全生产万无一失。

　　三、全面加强政府自身建设，为又快又好发展提供有力保障

　　面对新形势新任务，各级政府必须以“三个代表”重要思想和科学发展观为指导，坚持立党为公、执政为民，着力创新管理、转变职能，改进政风、真抓实干，不断提高行政效能和服务水平，更好地担负起站在新起点实现新发展的历史重任。

　　要始终做到与时俱进、开拓创新。着力建设学习型政府，在更高境界上解放思想，用科学发展、和谐发展的新理念指导工作。善于把握大势、据情决断，把上级部署与济宁实际结合起来，把长远目标与当前任务结合起来，把满腔热情与务实态度结合起来，创新发展思路与工作举措。健全重大问题调查研究制度、专家咨询制度、社会公示与听证制度和集体决策制度，完善决策后评估反馈机制，推进决策的科学化、民主化。

　　要始终做到依法行政、高效服务。自觉接受人大及其常委会的法律监督，认真贯彻执行各项决议决定，支持人民政协履行政治协商、民主监督和参政议政职能，认真办理人大代表建议、政协委员提案。深入落实《全面推进依法行政实施纲要》，善于运用法律手段管理经济社会事务，严格落实行政执法责任制、评议考核制和责任追究制。继续推进政府工作提速，提高行政审批效能和服务质量。认真贯彻《公务员法》，确立“支持改革、鼓励干事、保护创业”的理念，身体力行第一时间服务、零距离服务、全程服务，促进发展环境不断优化。

　　要始终做到求真务实、埋头苦干。牢固树立正确的政绩观，把全部精力用在求真务实、干事创业上。对年度任务目标、重点工作事项层层分解，明确责任，跟踪督查，严格考核。从市政府做起，进一步精简会议、文件，减少事务性活动，带头深入一线，到条件差、困难多、工作进展慢、群众不满意的地方攻坚克难、寻求突破。对坚持原则、敢抓善管、脚踏实地、勤勉敬业的干部，要关心再关心、支持再支持、保护再保护。

　　要始终做到执政为民、造福群众。坚持发展为了人民、发展依靠人民、发展成果由人民共享，实现好、维护好、发展好人民群众的根本利益。进一步办好市长公开电话，拓宽联系群众的渠道，尊重群众意愿，维护群众利益，增进群众福祉，从柴米油盐、衣食住行、入学就业、求医问药、助老托幼等方面入手，一件一件地为群众办实事、解忧难、谋利益。通过优质高效服务，让人民群众感受到党和政府的关怀与温暖。

　　要始终做到令行禁止、廉洁自律。深入落实学习党章、遵守党章、贯彻党章、维护党章的具体措施，切实强化党性修养，严格遵守政治纪律，与党中央和省、市委保持高度一致，不折不扣地执行国家的法律法规、方针政策。艰苦奋斗，勤俭节约，反对铺张浪费，带头创建节约型机关。建立健全教育、制度、监督并重的惩治和预防腐败体系，今年集中开展治理商业贿赂专项工作，重点治理工程建设、土地出让、产权交易、医药购销和政府采购等领域的商业贿赂问题，坚决纠正不正当交易行为，依法查处商业贿赂案件。继续纠正损害群众利益的不正之风。强化审计监督，支持监察机关依法查办案件，严惩腐败分子。

　　各位代表！新的目标催人奋进，新的征程任重道远。让我们在中共济宁市委的坚强领导下，高举邓小平理论和“三个代表”重要思想伟大旗帜，坚定信心，奋发图强，努力把“十一五”规划的宏伟蓝图变为美好现实，谱写济宁现代化建设的新篇章！
